# PD-1: Dual guard for immunopathology

**DOI:** 10.18632/oncotarget.4787

**Published:** 2015-07-05

**Authors:** Hye Ryun Kim, Hyo Jin Park, Sang-Jun Ha

**Affiliations:** Department of Biochemistry, College of Life Science & Biotechnology, Yonsei University, Seoul, Korea

**Keywords:** Immunology and Microbiology Section, Immune response, Immunity

Despite remarkable medical development, chronic infectious diseases caused by the infections with persistent pathogens such as hepatitis B virus (HBV), hepatitis C virus (HCV), human immunodeficiency virus (HIV), and *mycobacterium tuberculosis* (*M.tb*), have not been conquered yet. During the chronic infection, the interaction between host and chronic pathogen often fosters immune suppressive environment. Such environment leads to the exhaustion of pathogen-specific T cells, resulting in the failure to the eradication of pathogen but the success to the protection of host from immunopathological damage. Finally, host and pathogen ensure the reduction of host immunopathology but the extension of pathogen persistence.

**Figure 1:**
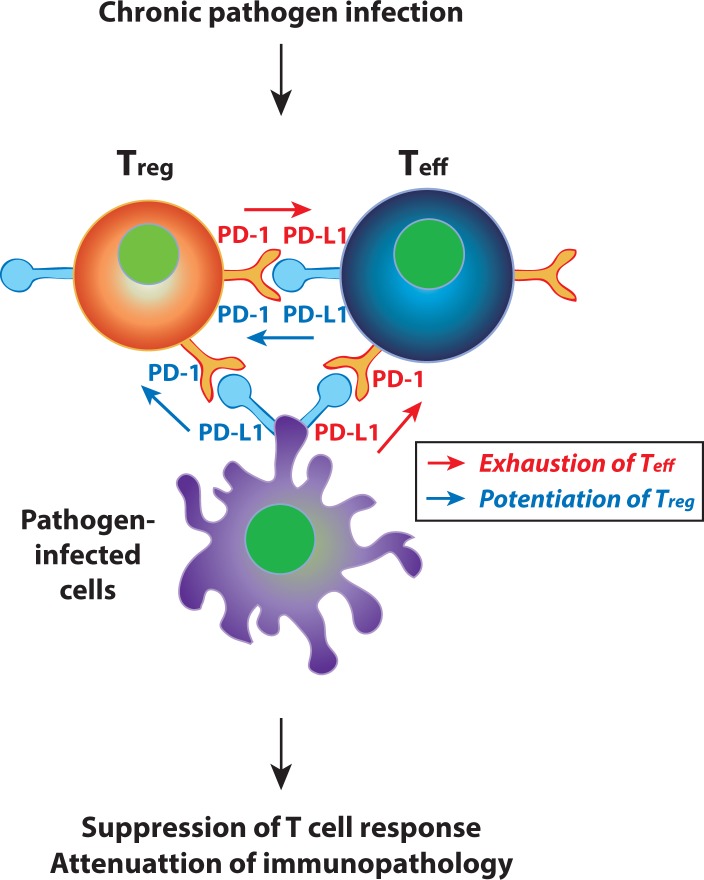
PD-1:PD-L1 pathway as a dual guard for the protection of immunopathological damage PD-1 is highly upregulated on T_reg_ cells as well as T_eff_ cells during chronic pathogen infection. PD-1:PD-L1 interactions between T_eff_ cells and pathogen-infected cells or between T_reg_ cells and T_eff_ cells provide Teff cell exhaustion signal or T_reg_ cell potentiation signal, thereby attenuating immunopathology.

Under the persistence of pathogens, effector T (T_eff_) cells lose progressively their effector function such as cytokine production and proliferative potential and finally become exhausted T (T_exh_) cells. T-cell exhaustion can be caused by various factors including immune checkpoints, cytokines, regulatory T (T_reg_) cells, and altered antigen presenting cells (APCs) [1]. Immune inhibitory receptors, also known as immune checkpoints, such as programmed death-1 (PD-1), T-cell immunoglobulin mucin 3 (TIM-3), cytotoxic T lymphocyte antigen-4 (CTLA-4), and lymphocyte activation gene-3 (LAG-3) are expressed on T_eff_ cells by T-cell receptor (TCR) stimulation and their expressions are maintained or even increased by repeated TCR stimulation during chronic infection. Upon ligands binding, such checkpoints are triggered to transmit inhibitory signal into T_eff_ cells by alone or their combination, which leads to the generation of T_exh_ cells and the attenuation of T-cell-mediated immunopathology. The interaction of PD-1 and its ligand, PD-L1, has been known to play a critical role in the exhaustion of T_eff_ cells. The role of PD-1 during chronic pathogen infection in suppressing T-cell function was demonstrated in the mouse model chronically infected with lymphocytic choriomeningitis virus (LCMV) [2]. This study showed that blockade of PD-1:PD-L1 interaction during chronic LCMV infection controlled viremia by restoring the function of virusspecific T_exh_ cells. More specifically, only PD-1^+^ CD8^+^ T cells, but not PD-1^−^ CD8^+^ T cells, regained their function upon PD-1:PD-L1 blockade, indicating that the effect of PD-1 on T-cell exhaustion is T-cell-intrinsic (Figure 1). Another interesting point was that PD-L1-deficient mice succumbed to chronic LCMV infection, resulting in death of mice within one week post-infection. PD-1:PD-L1 pathway seemed to be also critical in protecting mice from persistent bacterial infection because PD-1-deficient mice were extraordinarily sensitive to tuberculosis [3]. These data strongly suggests that PD-1:PD-L1 pathway regulates immune-mediated tissue damage during persistent infection by paralyzing the pathogen-specific T cells.

The claim that PD-1 expressed by T cells is a culprit of T-cell exhaustion is now generally accepted but there can be another causing factors. One of the candidates is T_reg_ cells, which play an important role for maintaining immunological self-tolerance and controlling T-cell-mediated autoreactive T-cell attack on tissue, because a number of studies showed the increase of T_reg_ cell population during persistent viral, helminthic, and bacterial infections. Depletion of T_reg_ cells in the mice chronically infected with LCMV was reported to strikingly expand functional LCMV-specific CD8^+^ T cells, suggesting the role of T_reg_ cells in the suppression of T-cell immune response during persistent pathogen infection [4]. However, this temporal ablation of T_reg_ cells failed to not only diminish viremia but promote immunopathology because it also triggered upregulation of PD-L1 on LCMV-infected cells, which again delivers negative signal to PD-1-expressing T cells. It has been widely known that T_reg_ cells constitutively express some immune checkpoints such as CTLA-4 and LAG-3. Of particular interest, like T_exh_ cells, T_reg_ cells during chronic pathogen infection was reported to further upregulated immune checkpoints including CTLA-4, LAG-3, PD-1, and TIM-3 [4, 5, 6]. However, while T_eff_ or T_exh_ cells-expressed immune checkpoints debilitate their effector function, T_reg_ cells-expressed ones, especially CTLA-4, have been reported to potentiate their suppressor function in direct or indirect way. For instance, CTLA-4 expressed by T_reg_ cells modulates dendritic cells (DCs), thereby enhancing T_reg_ cell suppressive function as positive regulator [7]. Taken together, albeit such a complexity, it is evident that T_reg_ cells can contribute to the inhibition of T-cell response and the protection of tissue from T-cell-mediated pathology.

In spite of the observation that PD-1 is upregulated on T_reg_ cells during chronic infection, it is less known about the role of PD-1 expressed by T_reg_ cells. We recently found that PD-1_hi_ T _reg_ cells generated during chronic LCMV infection displayed much stronger suppressive activity than PD-1_lo_ T_reg_ cells present in steady state [6]. Either PD-1 blockade on PD-1_hi_ T_reg_ cells or PD-L1 deficiency on T_eff_ cells dramatically ablated T_reg_ cell-mediated suppression of T_eff_ cell immune response, demonstrating the necessity of PD-1 on T_reg_ cells and PD-L1 on T_eff_ cells. These results highlight a critical role of PD-1:PD-L1 interaction between T_reg_ cells and PD-L1 on T_eff_ cells and define PD-1 upregulated on T_reg_ cells as a prerequisite for T_reg_ cell-mediated strong suppression (Figure 1). It should be further investigated for the molecular mechanism by which whether a ligation of PD-L1 in T_eff_ cells onto PD-1_hi_ T_reg_ cells triggers PD-1 signal in T_reg_ cells or a reverse signal via PD-L1 in T_eff_ cells upon a ligation of PD-1 provided by T_reg_ cells inhibits T_eff_ cell function. If the former is applied *in vivo*, PD-L1 is probably provided by pathogen-infected APCs as well as T_eff_ cells. Regarding cell-specific expression of PD-1, our results propose that PD-1 expressed by T_reg_ cells, in addition to that by T_exh_ cells, contributes to the functional suppression of T_eff_ cells and subsequent attenuation of immunopathology (Figure 1).

Collectively, PD-1:PD-L1 interaction is a major strategy for the suppression of T_eff_ cell-mediated immune response during chronic pathogen infection. Meanwhile, in regard to the immunopathological damage, PD-1:PD-L1 interaction protects T_eff_ cell-mediated tissue damage in the host. Of importance, PD-1:PD-L1 interaction is capable to occur in between T_reg_ cells and T_eff_ cells, in addition to between T_eff_ cells and pathogen-infected cells. In both case, T_eff_ cells become exhausted via direct interaction with separate partners and eventually fail to not only eradicate pathogens but also induce immunopathology in the host. Lastly, these studies provide perspectives regarding the practical and clinical treatment strategies for chronic infectious disease. Blockade of PD-1:PD-L1 can be useful to treat efficiently chronic virus infection but its potential risk for immunopathologic damage needs to be carefully monitored.
